# Long-Term Variability in Body Weight in Relation to the Risk of Dementia: A Prospective Cohort Study

**DOI:** 10.1093/geroni/igab046.197

**Published:** 2021-12-17

**Authors:** Hui Chen, Tianjing Zhou, Yuan Ma, Changzheng Yuan

**Affiliations:** 1 Zhejiang University, Hangzhou, Zhejiang, China (People's Republic); 2 Harvard T. H. Chan School of Public Health, Boston, Massachusetts, United States

## Abstract

The prospective association of body weight variability with dementia remains unclear. We aimed to investigate whether long-term variability in body weight is associated with the risk of late-life dementia and to explore their potential temporal relationship using data from a nationwide prospective cohort study of the United States. A total of 5,556 participants free of dementia in 2008 (55.66% women; mean [SD] age, 71.1 [3.1] years) were followed up to 8 years for doctor-diagnosed dementia reported biennially. Body weight variability was assessed as the coefficient of variation utilizing the body weight information collected over 16 years before 2008. Cox proportion hazard model was applied to estimate hazard ratio (HR) of dementia associated with body weight variability. Higher body weight variability was associated with an increased incidence of dementia after controlling for sociodemographic factors, lifestyle, mean body weight, and body weight change. The multi-variable adjusted HR of dementia of the highest quartile of body weight variability was 2.01 (95% CI 1.01-1.87) compared with the lowest. Every 1% increment in variability was associated with a 6.2% higher risk of dementia (HR=1.06, 95%CI 1.04,1.09, p-trend<0.001). Such association was observed for both Alzheimer’s disease and other types of dementia, with stronger association observed when body weight variability was assessed closer to dementia assessment.

